# Circulating levels of adiponectin and AdipoR expression in peripheral blood mononuclear cells are associated with lower respiratory tract Infection

**DOI:** 10.3389/fimmu.2024.1510760

**Published:** 2025-01-07

**Authors:** Qian Wang, Xuemei Wang, Danning Xu, Mengjie Jiang, Yidan Gao, Lijuan Jiang, Meilian Liu, Haoneng Tang, Lingli Tang

**Affiliations:** ^1^ Department of Laboratory Medicine, The Second Xiangya Hospital of Central South University, Changsha, Hunan, China; ^2^ Department of Biochemistry and Molecular Biology, University of New Mexico Health Sciences Center, Albuquerque, NM, United States

**Keywords:** adiponectin (APN), adiponectin receptor (AdipoR), lower respiratory tract, severity, infection, acute

## Abstract

**Objective:**

The role of adiponectin (APN) in regulating inflammation is well recognized in metabolic disease, but the dysregulation of APN in lower respiratory tract infection (LRTI) remains controversial. We aimed to measure APN and its signaling receptors, adiponectin receptor (AdipoR), in peripheral blood mononuclear cells (PBMCs) from LRTI patients to explore their potential roles in the LRTI process.

**Methods:**

A total of 99 LRTI patients from the Second Xiangya Hospital of Central South University were categorized into acute (n=35) and non-acute (n=64), and non-severe (n=62) and severe (n=37) groups. Serum APN was quantified using ELISA, and mRNA levels of PBMC AdipoRs were determined by RT-qPCR.

**Results:**

Both levels of APN in circulation and AdipoR1 mRNA were significantly elevated in the LRTI patients (*P*=2.61E-04; *P*=2.49E-08), while no statistical difference was observed for AdipoR2. APN levels were increased in the non-acute group compared to the acute group (*P*=6.06E-04) and AdipoR1 levels were higher in the severe group (*P*=0.004). Increased APN and AdipoR1 mRNA levels were positively associated with LRTI even after adjustment for sex, age, BMI and blood lipids (OR=1.10; 95% CI 1.04-1.18; *P*=9.61E-04; OR=2.69; 95% CI 1.29-5.58; *P*=0.008). Subgroup analyses based on sex, age, and BMI revealed APN elevation in males, ≥65-year-olds, and overweight individuals, with higher AdipoR2 mRNA in females and those under 65; AdipoR1 was uniformly elevated. Additionally, APN was negatively correlated with lymphocyte count in acute and severe subgroup; AdipoR1 was positively correlated with indicators of inflammation in LRTI group.

**Conclusion:**

Our study highlights that serum APN and AdipoR1 mRNA in PBMCs are associated with LRTI. Circulating APN and PBMC AdipoR1 have different significances in LRTI acute onset and severity.

## 1 Introduction

Lower respiratory tract infection (LRTI) has been a globally important public health issue, contributing to the burden of disease worldwide. LRTI ranks first among all types of infectious diseases in terms of both morbidity and mortality, according to the Institute for Health Measurement and Evaluation (IHME) in the Global Burden of Disease (GBD) study (1). In 2019, there were an estimated 257 million male and 232 million female LRTIs worldwide (2). In 2016, nearly 2.38 million people died from LRTI worldwide (3). The correlation between increased susceptibility to LRTI and worse clinical outcomes and obesity is not new (4), but adipose tissue, the largest endocrine organ in the body, has only recently been linked to its endocrine function and LRTI. Adipose tissue functions as a key endocrine organ by releasing a variety of biologically active substances (known as adipokines) with either pro- or anti-inflammatory properties (5) and adipokines have also been implicated in the dysregulated immune response in LRTI (6).

Adiponectin (APN) is a multifunctional hormonal protein secreted in large quantities by adipocytes (7) and can also be produced by other cells, including lymphocytes (8). Adiponectin receptor 1 (AdipoR1) and adiponectin receptor 2 (AdipoR2) are the two signaling receptors for APN and are expressed in lung epithelial cells, endothelial cells, and immune cells (9). AdipoRs are an emerging receptor family. APN has been shown to exert both anti-inflammatory and pro-inflammatory effects, depending on the disease (10). Previous studies have shown the insulin-sensitizing (11), anti-atherogenic, and anti-inflammatory effects of APN in metabolic diseases. Furthermore, various effects on different tissues were shown to be based on tissue-specific signaling pathways of APN (10). APN plays a pro-inflammatory role in a variety of autoimmune diseases such as rheumatoid arthritis (RA) (12) and inflammatory bowel disease (IBD) (13). Studies have shown that APN has anti-inflammatory activity in some inflammatory lung diseases. In patients with COPD (chronic obstructive pulmonary disease), serum APN levels are elevated, and in chronic obstructive pulmonary disease with acute exacerbation (AECOPD), levels are higher than in COPD (14).

In LRTI, there are fewer studies on APN, and especially AdipoR. Jiang Y et al. found increased levels of APN in the bronchoalveolar lavage fluid (BALF) of elderly patients with flu infection and concluded that APN aggravates flu infection (15). With the global spread of coronavirus disease 2019 (COVID-19) caused by severe acute respiratory syndrome coronavirus 2 (SARS-CoV-2), more studies have focused on adipokines and LRTI. SARS-CoV-2 may induce adipose tissue dysfunction, leading to adverse outcomes in acute COVID-19 (16), although some studies have shown unchanged or elevated APN levels (17), a majority of studies have reported decreased serum APN levels after COVID infection and agree in defining a clear role for APN in contributing to adverse outcomes in COVID-19 patients (18). In acute respiratory distress syndrome (ARDS), a severe form of LRTI, higher APN levels were associated with increased mortality among patients developing ARDS from extra-pulmonary etiologies (19). However, the study did not find a correlation between APN levels and disease severity or mortality of patients with ARDS overall. In conclusion, APN is complexly variable in LRTI and it is not clear whether it correlates with the acuity of onset as well as severity. As a traditional metabolic anti-inflammatory factor that is anti-inflammatory in COVID-19 but exacerbates infection in influenza, its exact role in LRTI also remains to be explored.

Respiratory defense against infection involves a diverse and complex system, and here are emerging principles of innate control of adaptive immunity (20). The emerging view is that APN is “a versatile player of innate immunity”. APN acts as a key regulator of the innate immune system and plays a major role in the progression of inflammation. APN senses metabolic stress and modulates metabolic adaptation by targeting the innate immune system under physiological and pathological conditions (21), and APN has been extensively studied for its immunomodulatory function in metabolic diseases (22). In the lung, the anti-inflammatory effects of APN are mainly observed in pneumocytes (9). Pang et al. (23) demonstrated that both AdipoR1 and AdipoR2 are also expressed on PBMC in healthy individuals, which implies that APN may regulate immune and inflammatory processes in a PBMC AdipoR-dependent signaling. However, after LRTI, the multiple roles of APN and PBMC AdipoR are not fully understood.

In summary, there is limited knowledge regarding the dynamics of APN levels, particularly the expression of its signaling receptors, in patients with LRTI. The objectives of this study were to (1) measure the serum APN levels and PBMC AdipoRs expression levels of LRTI patients, (2) reveal their distinct values in LRTI acute onset and severity, (3) investigate the levels of APN and AdipoRs across different genders, ages, and BMI, (4) analyze the correlation between APN, AdipoR and LRTI-related clinical indicators, and (5) explore the potential role of APN in the pathophysiological process of LRTI.

## 2 Materials and methods

### 2.1 Subjects

99 patients who were hospitalized and diagnosed with LRTI in the Second Xiangya Hospital of Central South University from August 2023 to May 2024 were selected. Inclusion criteria: (1) clinical diagnosis of LRTI-related diseases, including but not limited to community-acquired pneumonia, hospital-acquired pneumonia, chronic obstructive pulmonary disease with acute exacerbation, bronchiectasis with infection, and unspecified LRTI. (2) age >18 years. Subjects with the following conditions were excluded from this study: (1) body mass index (BMI) <18.5 or ≥28 kg/m^2^ (24) (Considering the small number of underweight (BMI <18.5 kg/m^2^) and obese (BMI≥28 kg/m^2^) patients enrolled, we excluded this part of the population to minimize interferences and allow better subgroup comparisons); (2) serum and whole blood samples were not sent for testing at the same time (± 1 day); (3) malignant tumors; (4) severe immunosuppression (e.g., hematologic disorders, AIDS, post-bone marrow transplantation); (5) severe cardiac, cerebral, renal, metabolic syndromes; (6) repeat cases.

**Figure 1:**
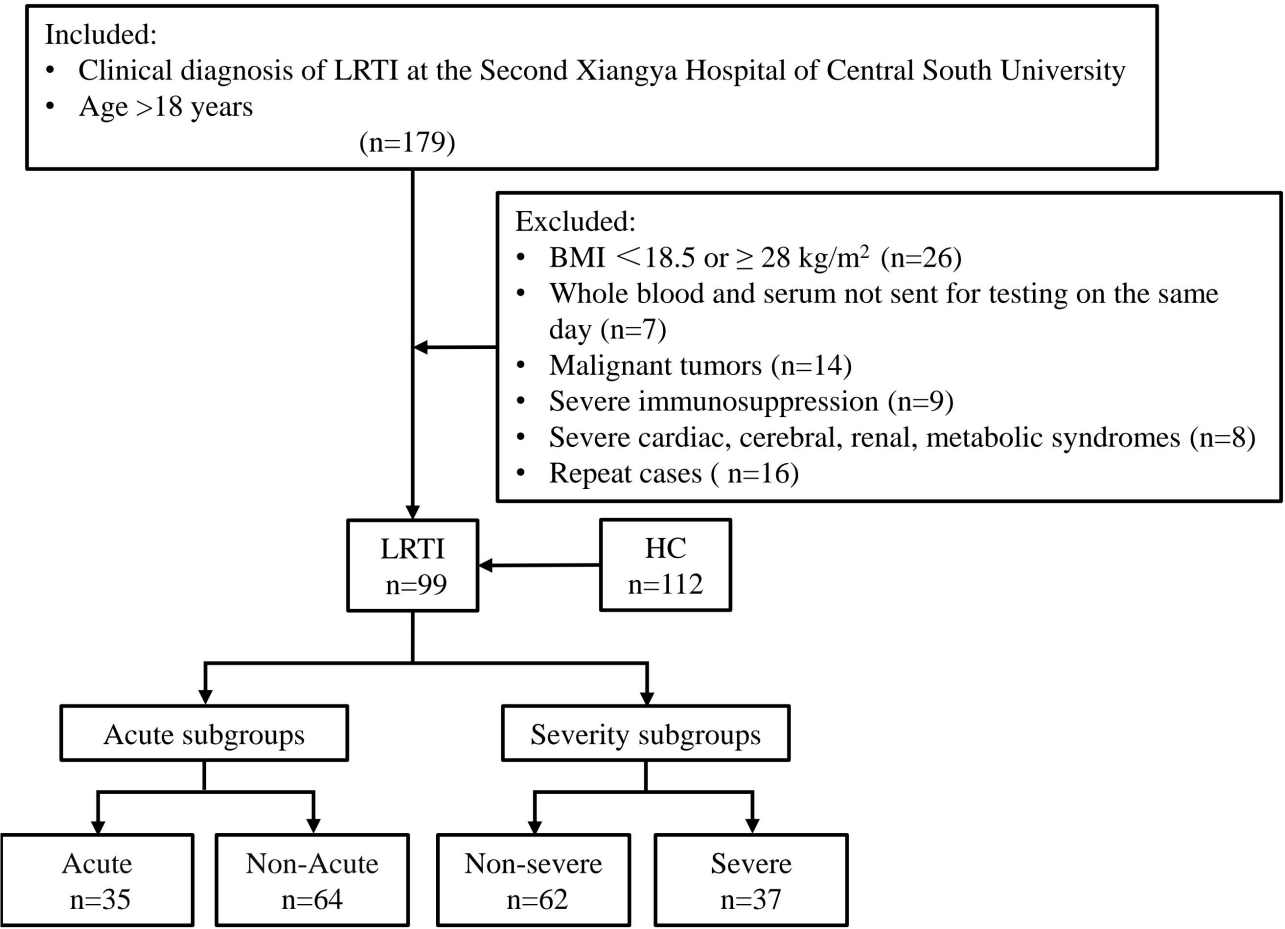
Schematic of study subject inclusion. The total number of subjects screened, the number of subjects excluded from each group, and the subgroups are shown. LRTI, lower respiratory tract infection; HC, healthy control.

112 healthy controls (HC) were sex- and age-matched individuals who underwent a physical examination at the same period in our health management center. The blood routine indicators related to infection and lipid metabolism were all within the 95% reference interval. The flow chart of patient enrollment is presented in **Figure 1**.

This study was conducted in accordance with the guidelines of the Declaration of Helsinki, and the study protocol was approved by the Ethics Committee of the Second Central South Xiangya Hospital (Ethics No. LYF20230156) and by the Chinese medical research registration information system (Ref number: MR-43-24-009374).

### 2.2 Definition of terms

Severe group, i.e., severe pneumonia group. The diagnosis was based on the 2019 American Thoracic Society (ATS)/Infectious Diseases Society of America (IDSA) Guideline (25). Primary criteria: (1) invasive mechanical ventilation is required; (2) vasoconstrictor therapy is required for infectious shock; minor criteria: (1) respiratory rate ≥30 breaths/min; (2) oxygenation index (PaO_2_/FiO_2_) ≤250; (3) multiple lobar infiltrates; (4) hypothermia (T<36℃); (5) leukopenia (WBC<4.0×10^9^/L); (6) thrombocytopenia (platelets <10.0×10^9^/L); (7) hypotension, requiring forceful fluid resuscitation; (8) impaired consciousness/disorientation; (9) azotemia (BUN≥20mg/dL). A diagnosis of severe pneumonia is made if 1 major criterion or 3 minor criteria or more are met. The non-severe pneumonia group was defined as patients who had a clear clinical diagnosis of LRTI but did not fit into the severe pneumonia group.

Acute onset criteria: after the patient was definitively diagnosed with LRTI, the patient’s respiratory history was retrospectively reviewed up to the time of specimen collection, referred to as the duration of illness. Respiratory history encompassed symptoms and/or signs. Respiratory symptoms included cough, sputum production, dyspnoea, wheeze or chest discomfort/pain and no alternative explanation; respiratory signs were ascertained through lung imaging. An acute illness (present for 21 days or less), usually with cough as the main symptom, with at least one other lower respiratory tract symptom (26).

Normal weight: 18.5≤ BMI <24 kg/m^2^; overweight:24≤ BMI <28 kg/m^2^.

### 2.3 Data collection

Retrospective clinical indicators were collected during the current hospitalization. BMI was calculated from height and weight (weight ÷ height^2^, kg/m^2^). All blood samples were collected in the morning (7-8 am) after 10-12 hours of overnight fasting. Serum samples (centrifugation: 3000g for 5 minutes) or whole blood samples were used for subsequent testing as needed. Some parameters of routine blood counts (i.e., leukocytes, neutrophils, lymphocytes, eosinophils, and monocytes) were measured by a Sysmex XN-20 automated hematology analyzer (Sysmex Corporation, Japan). Triglycerides (TG), total cholesterol (TC), high-density lipoprotein cholesterol (HDL-C), and low-density lipoprotein cholesterol (LDL-C) were measured by a HITACHI 7600 automated analyzer (Hitachi, Japan). C-reactive protein (CRP) was measured by a Beckman IMMAGE 800 fully automated analyzer system (Beckman Coulter Co., Ltd., China). Procalcitonin (PCT) was measured using Roche Cobas e801 (Roche, Germany). Erythrocyte sedimentation rate (ESR) was measured by monitor100 hemosiderometer. D-Dimer was measured with a STAGO hemagglutination meter. Lymphocyte subpopulation results were measured using a BD FACSCanto II flow cytometer (Becton, Dickinson and Company, USA). Immunoglobulins IgG, IgA, IgM, and IgE were measured using Roche C702 automatic biochemistry analyzer (Roche, Germany). Information on the measurement methods of the above parameters is provided in Additional file 1: **Supplementary Table S1** and all indicators were measured under standardized conditions in an ISO 15189 accredited medical laboratory.

### 2.4 APN detection

The serum was collected and immediately stored at -80°C until analysis. Serum samples were measured by enzyme-linked immunosorbent assay (ELISA) within 6 months, detected at 8000-fold dilution according to the manufacturer’s instructions (Elabscience^®^ Biotechnology, E-EL-H6122, China), plotted on a calibration curve (R^2^ > 0.99), and then quantified.

### 2.5 AdipoR measurement

PBMC isolation was performed as soon as possible on the same day after the collection of fresh whole blood samples, and the method used was Ficoll-Paque (Mead Pacific (Tianjin) Biotechnology Co.) density gradient centrifugation. Freshly extracted PBMCs were subjected to total RNA extraction using Trizol reagent (Sangon Biotech, B511321) according to the manufacturer’s guidelines. Total RNA was reverse transcribed into complementary DNA (cDNA) using the PrimeScript RT Reagent Kit (TaKaRa Bio, RR047B) according to the manufacturer’s instructions. Quantitative polymerase chain reaction (qPCR) was performed using SYBR Green Premix Ex Taq II (TaKaRa Bio, RR820A) according to the manufacturer’s instructions. hTubulin was used as an internal control. The primer sequences used were: AdipoR1 F: CTGGCTAAAGGACAACGACTA; AdipoR1 R: TGTATGAATGCGGAAGATGCT; AdipoR2 F: CTGTCTTGGTAAGCCTGGATGTG, AdipoR2 R: GCTGACAACTCCGTACTACAACTG; hTubulin F: CTGGACCGCATCTCTGTGTACT; hTubulin R: GCCAAAAGGACCTGAGCGAACA, primer purification method was HAP, and all samples were assayed by setting up 3 replicate wells. qPCR was performed on a Roche LightCycler 96 PCR Detection System (Roche, Switzerland) according to the manufacturer’s protocol. The thermal cycling protocol consisted of an initial pre-denaturation step at 95°C for 30 seconds (s), followed by 50 denaturation cycles at 95°C for 5 s, annealing at 60°C for 30s, and finally a dissociation procedure. Relative quantification of each sample was performed by calculating 2^-ΔΔCq^.

### 2.6 Statistical analysis

Categorical data were expressed as numbers and percentages, while continuous variables were expressed as median and interquartile range or mean and standard deviation, depending on the distribution. Spearman’s rank correlation coefficient was used to analyze the possible association of APN and AdipoR with other clinical indicators. Statistical tests including the nonparametric Mann-Whitney U test, nonparametric Kruskal-Wallis test, chi-squared test, t-test and one-way ANOVA test were performed using IBM SPSS Statistics 26.0 software. *P* values <0.05 were considered statistically significant.

## 3 Results

### 3.1 Baseline characteristics of the participants

The baseline characteristics of the subjects are shown in **Table 1**. At baseline, there were no significant differences in gender between the HC and LRTI and subgroups, with a higher proportion of males in each group (70.54% in the HC group, 69.70% in the LRTI group). The overall age of the study population was high and was highest in the non-acute group (71.00[64.25,77.00]). BMI was lower in the LRTI group than in the HC group (*P*=6.30E-04), with no significant difference between LRTI subgroups. Except for eosinophil count, each index showed a significant difference between the LRTI and the HC group. The LRTI group had higher WBC, neutrophil, monocyte count and TG, and lower lymphocyte count, TC, HDL-C, and LDL-C. In the severity subgroups, neutrophil count was higher, and lymphocyte count, TC, HDL-C, and LDL-C were lower in the severe group. In the subgroups divided by acute onset of LRTI, HDL-C was significantly lower in the acute group than in the non-acute group.

**Table 1:** Baseline characteristics of the HC and LRTI group and subgroups.

Characteristic	HC and LRTI groups			Severity subgroups			Acute subgroups		
	HC (n=112)	LRTI (n=99)	*P* ^a^	Non-severe (n=62)	Severe (n=37)	*P* ^b^	Acute (n=35)	Non-acute (n=64)	*P* ^c^
Sex			0.894			0.584			0.524
Male	79 (70.54%)	69 (69.70%)		42 (67.74%)	27 (72.97%)		23 (65.71%)	46 (71.87%)	
Female	33 (29.46%)	30 (30.30%)		20 (32.26%)	10 (27.03%)		12 (34.29%)	18 (28.13%)	
Age (years)	66.00 [58.00,74.75]	69.00 [60.00,77.00]	0.149	70.50 [61.25,77.00]	67.00 [58.00,77.00]	0.452	64.00 [49.00,75.00]	71.00 [64.25,77.00]	**0.022**
BMI (kg/m^2^)^d^	23.70±2.30	22.42±2.40	**6.30E-04**	22.42±2.34	22.43±2.54	0.988	22.68±2.58	22.29±2.31	0.488
Routine blood tests									
WBC count(10^9^/L)	5.51 [4.89,6.51]	7.93 [6.49,11.83]	**1.33E-15**	7.61 [6.28,10.14]	9.49 [6.53,12.25]	0.149	9.96 [6.31,12.24]	7.66 [6.49,10.56]	0.302
neutrophil count(10^9^/L)	3.08 [2.67,3.86]	6.34 [4.77,9.19]	**0.00E+00**	5.84 [4.13,8.28]	8.16 [5.14,10.71]	**0.021**	7.46 [5.27,10.43]	6.02 [4.54,8.98]	0.180
lymphocyte count(10^9^/L)	1.81 [1.59,2.14]	1.04 [0.67,1.45]	**0.00E+00**	1.22 [0.79,1.70]	0.84 [0.58,1.14]	**4.10E-04**	1.06 [0.76,1.37]	1.01 [0.64,1.69]	0.939
eosinophil count(10^9^/L)	0.13 [0.08,0.20]	0.12 [0.03,0.19]	0.726	0.13 [0.05,0.22]	0.09 [0.01,0.18]	0.200	0.07 [0.01,0.17]	0.13 [0.05,0.21]	0.189
mononuclear cell count(10^9^/L)	0.33 [0.28,0.43]	0.50 [0.33,0.64]	**9.82E-07**	0.48 [0.33,0.63]	0.51 [0.30,0.65]	0.919	0.51 [0.33,0.63]	0.47 [0.32,0.70]	0.921
Blood lipids^e^									
TG(mmol/L)	1.23 [0.95,1.50]	1.35 [0.96,1.88]	**0.043**	1.23 [0.95,1.64]	1.59 [0.95,2.49]	0.115	1.40 [1.03,2.07]	1.35 [0.92,1.88]	0.581
TC(mmol/L)	4.53 [3.94,4.83]	3.74 [3.01,4.49]	**7.60E-05**	4.07±1.15	3.32±1.46	**0.010**	3.68±1.28	3.79±1.38	0.873
HDL-C(mmol/L)	1.30 [1.21,1.47]	0.84 [0.56,1.08]	**0.00E+00**	0.96 [0.79,1.19]	0.62 [0.43,0.91]	**3.46E-04**	0.66 [0.44,0.99]	0.89 [0.64,1.18]	**0.048**
LDL-C(mmol/L)	2.54±0.61	2.26±1.04	**0.019**	2.45 [1.87,3.07]	1.55 [1.15,2.62]	**0.002**	2.19 [1.53,3.02]	2.23 [1.70,2.82]	0.934

Data are presented as mean ± SD or median [IQR, Q1-Q3] for continuous variables and as absolute numbers and percentages for categorical variables. HC, healthy control; LRTI, lower respiratory tract infection; BMI, body mass index; WBC, white blood cell; TG, triglyceride; TC, total cholesterol; HDL-C, high-density lipoprotein-cholesterol; LDL-C, low-density lipoprotein-cholesterol.

^a b c^ Comparisons between the two groups were performed using χ^2^ test or t-test or nonparametric test. ^d e^ Missing data, the actual sample size is ^d^ HC: 85, LRTI: 79, ^e^ LRTI: 68. Statistically significant values are identified in boldface.

### 3.2 The increased levels of APN and AdipoR1 mRNA in patients with LRTI

**Figure 2:**
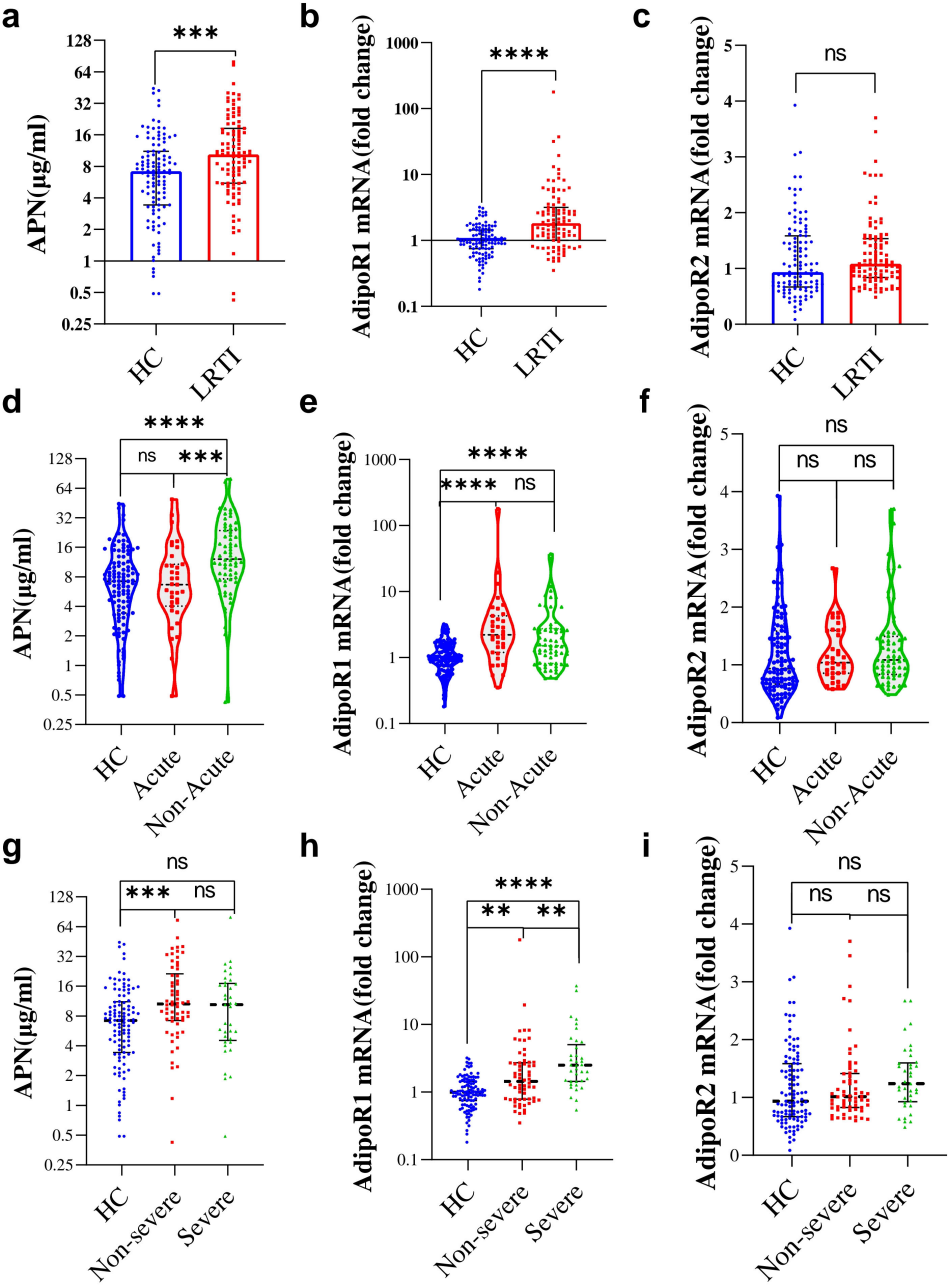
Serum APN, PBMC AdipoR1 and AdipoR2 mRNA levels in HC and LRTI groups and subgroups. **(A-C)** Box scatter plots showing **(A)** serum APN, **(B)** PBMC AdipoR1 mRNA, **(C)** PBMC AdipoR2 mRNA levels in HC and LRTI patients; d-f Violin plots showing **(D)** serum APN, **(E)** PBMC AdipoR1 mRNA, **(F)** PBMC AdipoR2 mRNA levels in HC and acute subgroups; **(G–I)** String plots showing **(G)** serum APN, **(H)** PBMC AdipoR1 mRNA, **(I)** PBMC AdipoR2 mRNA levels in the HC and severity subgroups. **(A–C)** data were analyzed by Mann-Whitney U test, **(D–I)** data were analyzed by Kruskal-Wallis. *P<0.05, **P<0.01, ***P<0.001, ****P<0.0001, ns, not significant.

To investigate the correlation between APN and AdipoRs and lung infection, we examined the circulating APN levels in the HC and LRTI groups. Additionally, we isolated PBMCs and then performed RT-qPCR for AdipoRs mRNAs. The results are shown in **Figure 2**. The circulating levels of APN (10.56[5.52,18.68] vs. 7.21[3.42,11.01] ug/ml, *P*=2.61E-04, **Figure 2A**) and the mRNA levels of AdipoR1 in PBMCs (1.79[0.99,3.04] vs. 0.99[0.75,1.44], *P*=2.49E-08, **Figure 2B**) were higher in LRTI patients than in the HC group, but PBMC AdipoR2 mRNA levels were not higher in LRTI group (*P*=0.078, **Figure 2C**). In acute subgroups, APN levels were higher in non-acute group compared to the acute group (*P*=6.06E-04, **Figure 2D**); PBMC AdipoR mRNA levels did not differ significantly (**Figures 2E, F**). Among the severity subgroups, AdipoR1 mRNA levels were higher in the severe group than in the non-severe group (2.50[1.44,5.03] vs. 1.36[0.78,2.70], *P*=0.004, **Figure 2H**), and APN and AdipoR2 mRNA levels were not statistically different (**Figures 2G, I**).

### 3.3 Association of LRTI with serum APN levels and PBMC AdipoRs mRNA levels

**Figure 3:**
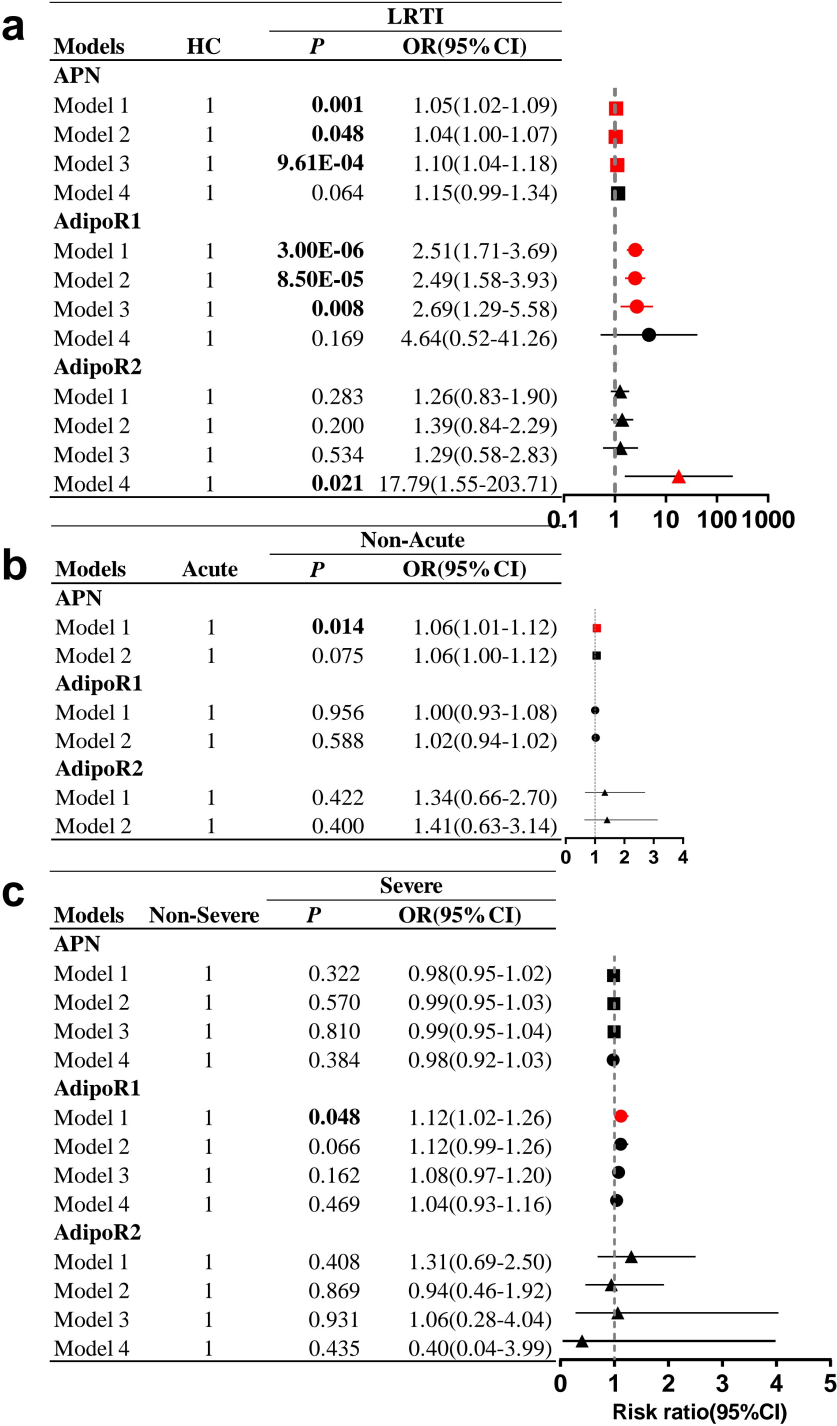
Forest plot of HC, LRTI and subgroups. Multifactorial logistic regression analyses in **(A)** LRTI and HC, **(B)** acute subgroups, **(C)** severe subgroups. All pairwise comparisons were made with the first group as the reference. **(A)** Model 1 was unadjusted; model 2 was adjusted for sex, age and BMI; model 3 was adjusted based on model 2 by adding TG, TC, HDL-C, and LDL-C; and model 4 was adjusted based on model 3 by adding WBC count, neutrophil count, lymphocyte count, and monocyte count. **(B)** Model 1 was unadjusted; model 2 was adjusted for sex, age, BMI and HDL-C. **(C)** Model 1 was not adjusted; model 2 was adjusted for sex, age and BMI; model 3 was adjusted based on model 2 by adding TC, HDL-C, and LDL-C; and model 4 was adjusted on the basis of model 3 by adding neutrophil and lymphocyte count. OR, odds ratio; CI, confidence interval. Statistically significant values are shown in bold.

Based on the results of univariate analyses between the HC and LRTI groups and subgroups (**Table 1**), factors with significant influences (*P*<0.05) were included in multivariate logistic regression analyses to explore the confounding factors of APN and AdipoRs between the LRTI and subgroups (**Figure 3**).

In the multivariate model adjusted for sex, age, BMI, and blood lipids (model 3, **Figure 3A**), LRTI was significantly associated with increased serum APN levels (OR=1.10; 95% CI 1.04-1.18; *P*=9.61E-04) and elevated PBMC AdipoR1 mRNA levels (OR=2.69; 95% CI 1.29-5.58; *P*=0.008), but after further adjustment for WBC, neutrophil, lymphocyte and monocyte counts (model 4, **Figure 3A**), there was no significant difference in APN and PBMC AdipoR1 mRNA levels between the HC and LRTI groups. In unadjusted subgroup analyses (model 1, **Figure 3B**), increased APN levels were associated with the non-acute group compared to the acute group (OR=1.06, 95% CI 1.01-1.12, *P*=0.014). Additionally, increased PBMC AdipoR1 levels were associated with severe disease (OR=1.12; 95% CI 1.02-1.26; *P*=0.048) (model 1, **Figure 3C**). However, after adjustment for potential confounders including sex, age, and BMI (model 2, **Figures 3B, C**), there was no significant correlation between these subgroups and either APN or PBMC AdipoR1 mRNA levels.

### 3.4 APN and AdipoR1/2 in LRTI patients across sexes, ages, and BMI categories

**Figure 4:**
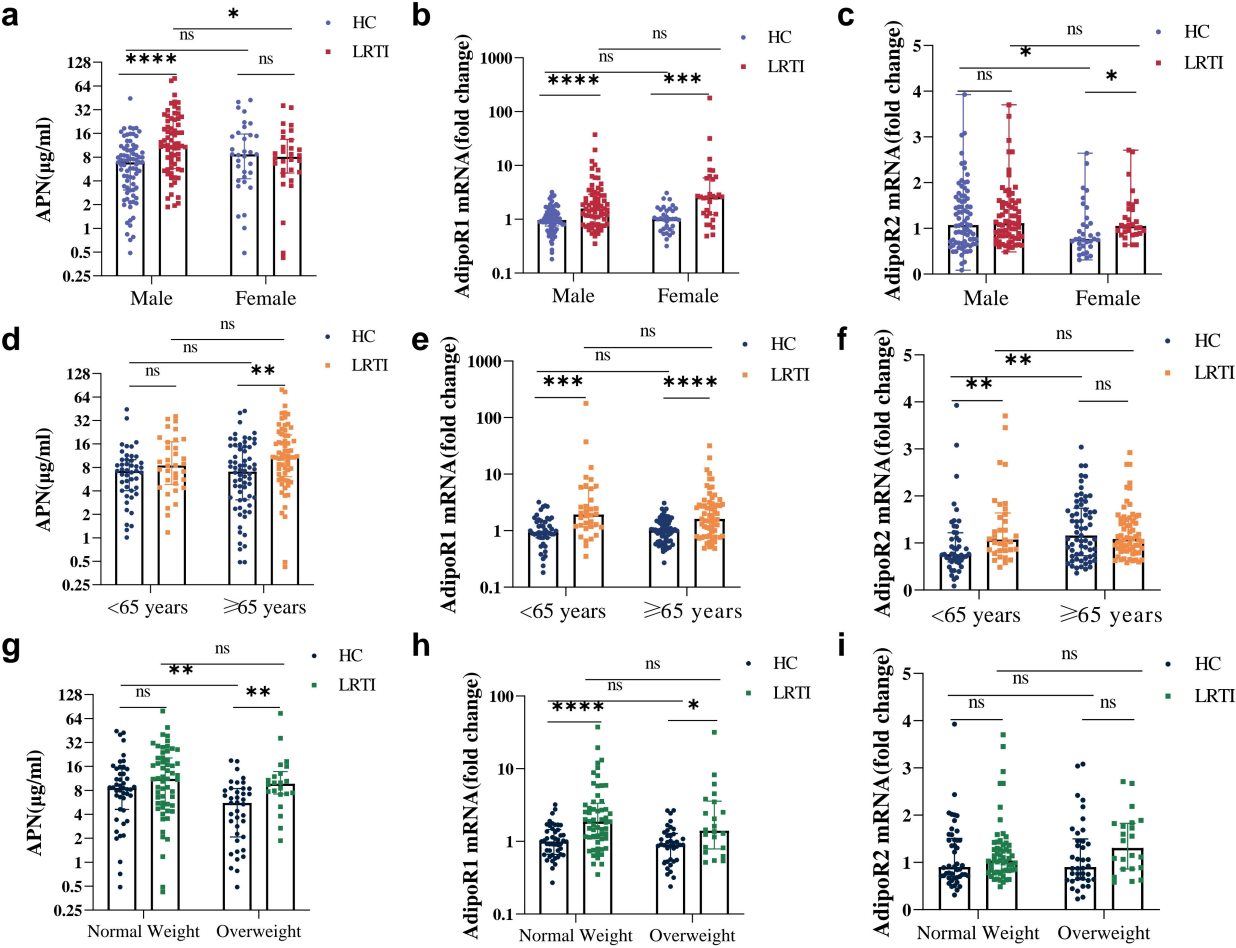
Serum APN, PBMC AdipoR1 and AdipoR2 mRNA levels in HC vs. LRTI at different sexes, ages and weights. Comparison of **(A, D, G)** serum APN, **(B, E, H)** PBMC AdipoR1 mRNA, **(C, F, I)** PBMC AdipoR2 mRNA for HC and LRTI at different **(A–C)** sexes, **(D–F)** ages, **(G–I)** body weights. Data were analyzed by Mann-Whitney U test. *P<0.05, **P<0.01, ***P<0.001, ****P<0.0001, ns, not significant.

We further explored changes in APN and its receptors in LRTI patients of different genders, ages, and BMIs. In the HC group, APN levels were decreased in overweight individuals (**Figure 4G**); AdipoR2 mRNA was lower in females (**Figure 4C**) and those <65 years of age (**Figure 4F**). After infection, APN was significantly increased only in males (**Figure 4A**), in individuals aged 65 years and above (**Figure 4D**) and in overweight individuals (**Figure 4G**); AdipoR2 mRNA levels were higher in females (**Figure 4C**) and individuals under 65 years of age (**Figure 4F**), but not significantly elevated across any BMI subgroups (**Figure 4I**). Noteworthy, AdipoR1 was not affected by sex, age, or weight (**Figures 4B**, **E**, **H**), which was elevated in all subgroups after infection.

### 3.5 Correlation between APN circulating levels, AdipoRs expression levels and clinical outcomes in HC, LRTI group and subgroups

**Figure 5:**
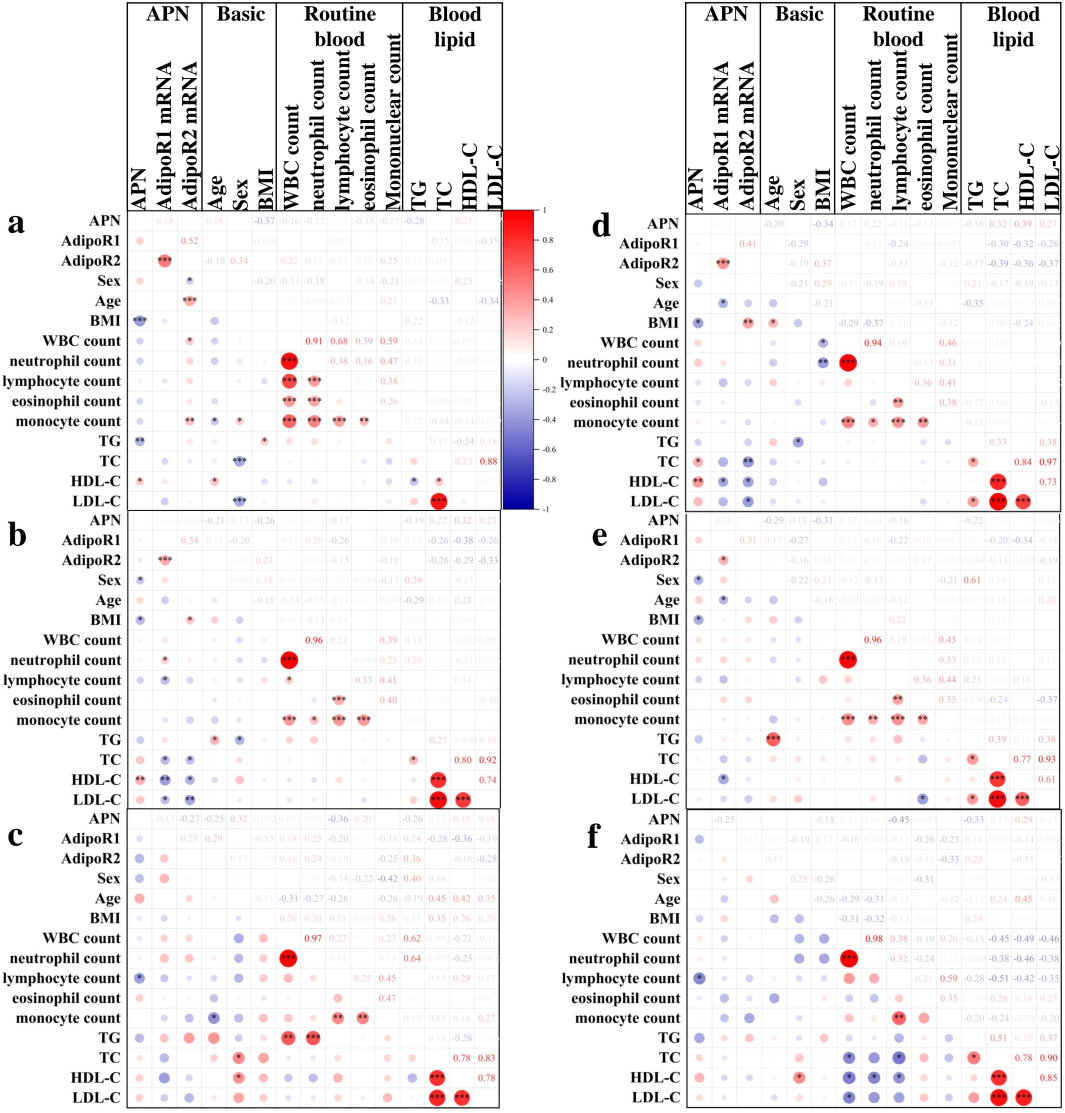
Correlation analysis of APN and AdipoR with clinical outcomes in HC and LRTI groups. Heatmap of Spearman’s correlation between serum APN, PBMC AdipoR1 mRNA, PBMC AdipoR2 mRNA and clinical indicators in **(A)** HC, **(B)** LRTI, **(C)** acute, **(D)** non-acute, **(E)** non-severe, and **(F)** severe group. *P<0.05, **P<0.01, ***P<0.001.

Correlation analysis showed that in the HC group (**Figure 5A**), serum APN was significantly correlated with BMI (*P*=4.40E-04, r=-0.37), TG (*P*=0.003, r=-0.28), HDL-C (*P*=0.028, r=0.21), and PBMC AdipoR2 mRNA was correlated with sex (*P*=0.049, r=-0.19), age (*P*=2.6E-4, r=0.34), WBC (*P*=0.023, r=0.22), and monocyte count (*P*=0.008, r=0.25); in the LRTI group (**Figure 5B**), APN was associated with sex(*P*=0.034, r=-0.21), BMI (*P*=0.019, r=-0.26), HDL-C (*P*=0.008, r=0.32), and PBMC AdipoR1 mRNA with neutrophil count (*P*=0.047, r=0.20), lymphocyte count (*P*=0.011, r=-0.26), TC (*P*=0.031, r=-0.26), HDL-CH (*P*=0.002, r=-0.38), LDL-CH (*P*=0.035, r=-0.26) were significantly correlated. In the acute group (**Figure 5C**), APN was negatively correlated with lymphocyte count (*P*=0.033, r=-0.36); in the non-acute group (**Figure 5D**), APN was correlated with BMI (*P*=0.013, r=-0.34), TC (*P*=0.030, r=0.32), HDL-C (*P*=0.008, r=0.39); PBMC AdipoR1 mRNA was correlated with age (*P*=0.019, r=-0.29), HDL-C (*P*=0.033, r=-0.32); PBMC AdipoR2 mRNA was correlated with BMI (*P*=0.007, r=0.37), TC(*P*=0.009, r=-0.39), HDL-C (*P*=0.008, r=-0.36), LDL-C (*P*=0.012, r=-0.37). In the non-severe group (**Figure 5E**), serum APN was correlated with sex (*P*=0.025, r=-0.29) and BMI (*P*=0.027, r=-0.31) and PBMC AdipoR1 mRNA was correlated with age (*P*=0.035, r=-0.27) and HDL-C (*P*=0.039, r=-0.34); in the severe group (**Figure 5F**), APN was negatively correlated with lymphocyte count (*P*=0.009, r=-0.42).

### 3.6 Further exploration of the correlation of APN and PBMC AdipoRs in LRTI

**Figure 6:**
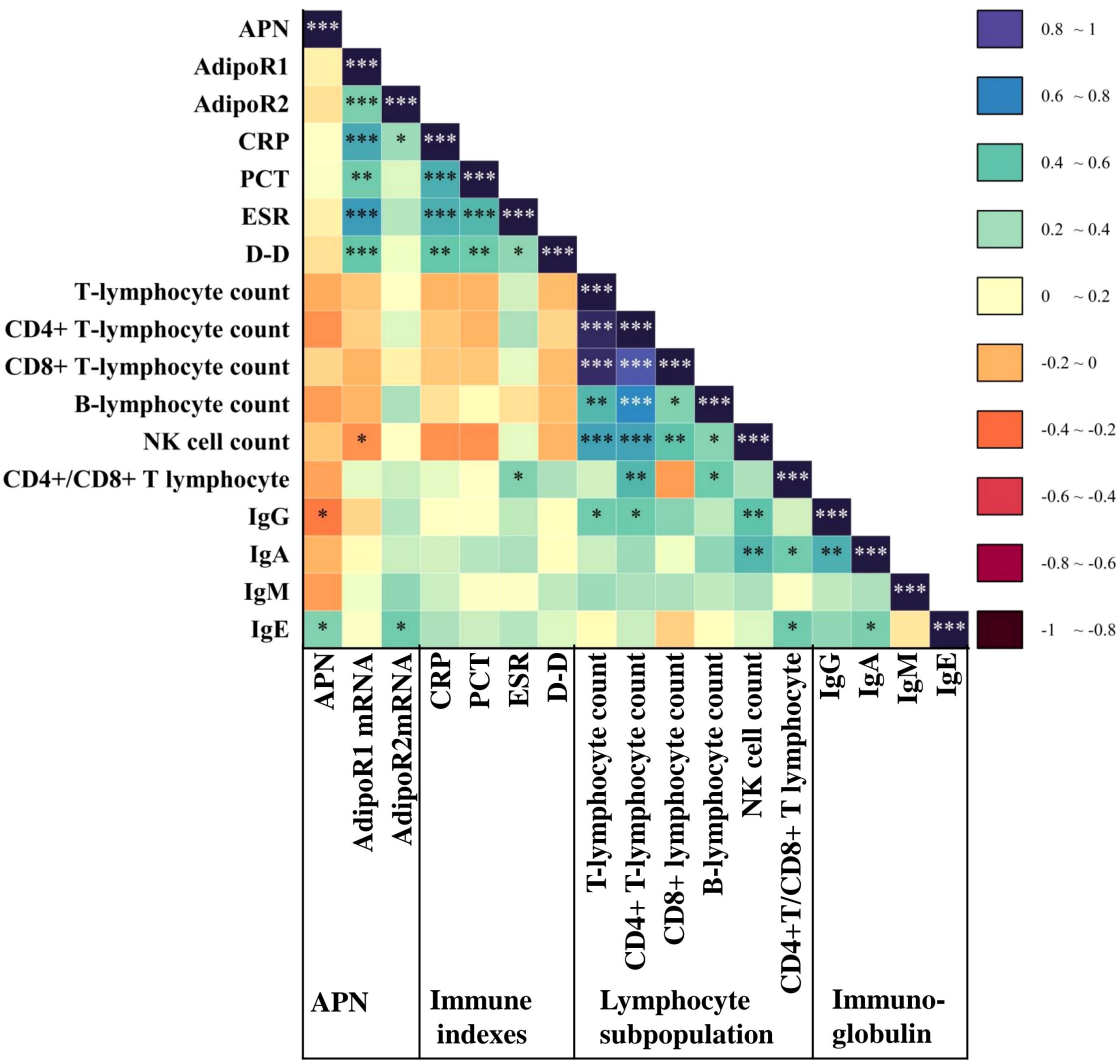
Correlation of APN and AdipoR with inflammatory markers, lymphocyte subpopulation and immunoglobulins in the LRTI group. Heatmap of serum APN, PBMC AdipoR1 mRNA, and PBMC AdipoR2 mRNA levels in the LRTI group in correlation with inflammatory markers, lymphocyte subpopulations, and immunoglobulin Spearman. *P<0.05, **P<0.01, ***P<0.001. CRP, C-reactive protein; PCT, procalcitonin; ESR, erythrocyte sedimentation Rate; D-D, D dimer; Ig, immunoglobulin.

We also performed a correlation analysis to assess the relationship between APN and its signaling receptors with inflammatory indicators, lymphocyte subsets, and immunoglobulins in LRTI patients. Our analysis (**Figure 6**) revealed that APN was correlated with IgG (*P*=0.015, r=-0.35) and IgE (*P*=0.041, r=0.31). PBMC AdipoR1 mRNA exhibited a positive correlation with CRP (*P*=5.10E-06, r=0.34), PCT (*P*=0.004, r=0.49), ESR (*P*=3.60E-07, r=0.35) and D-D (*P*=9.60E-04, r=0.52), and a negative correlation with absolute NK cell counts (*P*=0.049, r=-0.29). Additionally, PBMC AdipoR2 mRNA was positively correlated with CRP (*P*=0.038, r=0.24) and IgE (*P*=0.019, r=0.36).

## 4 Discussion

LRTI ranks high among all types of infectious diseases in terms of both morbidity and mortality (1–3). APN has been extensively studied in metabolic diseases (10). There is increasing data suggesting that APN exerts an anti-inflammatory effect in the lung (9). However, APN, in particular AdipoR, has not been well studied in pulmonary infection. We confirmed that APN and its signaling receptor AdipoR1 in PBMCs are associated with LRTI.

Our study revealed that APN serum levels were significantly elevated in the LRTI group compared with the HC group, even after adjustment for sex, age, BMI and blood lipids. Change levels and role of APN in respiratory infectious diseases are inconclusive. Our study is consistent with the results of influenza and AECOPD and differs from the results of COVID-19. Consistently, Jiang Y et al. found that elevated levels of APN were found in BALF of elderly patients diagnosed with influenza infection, and that mRNA and protein expression of APN were higher in lung histiocytes than in matched young control cells, ultimately finding that APN exacerbates influenza infection in the elderly via IL-18 (15). APN serum levels were increased in patients with COPD (14, 27, 28), and levels were higher in AECOPD (28). APN levels in COVID-19 patients are not consistent. Tonon F et al. found no significant difference in APN serum levels between COVID-19 pneumonia patients (n=48) and age-, sex-, and BMI-matched healthy person (n=36) (29); S-M Kearns et al. found that COVID-19 respiratory failure patients had lower levels of APN (n=12), even after adjustment for age, sex, BMI and other covariates (30), and Perrotta F et al. found lower levels of APN in patients with severe COVID-19 (n=62) compared to age- and sex-matched HC (31). The above results suggested that APN is associated with acute and non-acute lung inflammation, which may be influenced by the history of respiratory disease. We found the non-acute group had increased circulating APN levels compared to the acute group. Our study elucidated the role of APN levels in the non-acute onset of respiratory infection. We speculate that elevated APN may play a proinflammatory role in non-acute lung inflammation by promoting the secretion of cytokines such as IL-18.

In contrast, our study shows that APN serum level did not differ significantly between the non-severe and severe groups. There is also inconsistency in whether APN correlates with COVID-19 severity. APN levels were similar in mild, moderate, and severe groups classified on the basis of hospitalization requirements (32), and in subgroups classified on post-hospitalization outcomes, APN was significantly different only in non-obese individuals (17). More studies have shown APN to be associated with COVID severity, with Hindsberger B et al. finding that serum APN levels on admission were negatively associated with mortality and respiratory failure in COVID-19 hospitalized patients (n=123) (33); Flikweert AW et al. found lower APN levels in severe (the need for oxygen support, n=159) and critical patients(the need for mechanical ventilation and other organ support, n=71), compared to those that did not require hospitalization (n=30) (34); V Pavel found serum APN levels in severe COVID-19 patients (n=60) were lower than in those with moderate disease (n=64) (35); but A Ismaiel et al. found by meta-analysis that APN serum levels were increased in patients with severe compared to mild COVID-19 (36); Mester P et al. in a study of 156 patients with SIRS/sepsis found that plasma APN levels were higher in 18 non-surviving patients than in 84 surviving patients (37). Based on these complex results, we speculate that the secretion level of APN, an adipokine, is influenced by a variety of factors, including sex, age, BMI (37), and disease specificity.

Our results show that PBMC AdipoR1 mRNA levels are elevated in LRTI patients, especially in severe patients. To the best of our knowledge, our study demonstrates for the first time the value of PBMC AdipoR and AdipoR1 alone in differentiating severe from non-severe pneumonia. For the elevation of PBMC AdipoR, we speculate the possible reasons. Compared with the HC group where AdipoR1 was associated with monocyte count, in the LRTI group AdipoR1 was associated with lymphocyte count, this shift led us to hypothesize that the elevation of PBMC AdipoR1 was mainly related to the elevated expression of lymphocyte AdipoR genes. Furthermore, the positive correlation results between AdipoR1 and inflammatory indicators CRP, PCT, ESR and D-D also led us to hypothesize that AdipoR1 is more likely to play a pro-inflammatory role in LRTI. From our result, PBMC AdipoR1 plays a more important role in LRTI than AdipoR2. Few studies have been conducted on the comparison of AdipoR1 and AdipoR2. S Zhang et al. found that the protective effects of APN/AdipoR signaling against brain injury are AdipoR1 dependent, not AdipoR2 (38); Jiang Y et al. in influenza-infected AdipoR1 and 2 knockout mice, found that only AdipoR1^-/-^ aged mice exhibited less severe symptoms of infection after influenza infection (15). We infer that AdiopR1 rather than AdiopR2, plays a more important role in respiratory infectious diseases. AdipoR1 is associated with AMPK (5’-adenosine monophosphate-activated protein kinase) signaling pathway activation (10), which needs to be further investigated in LRTI.

In our study, we found that APN was significantly higher only in men, individuals aged 65 years and above, and those with overweight status in the infected group, and AdipoR2 was higher in women and individuals under 65 years of age, while AdipoR1 levels were unaffected by these demographic factors and increased across all subgroups, suggesting it may serve as a reliable marker of LRTI. Our findings are consistent with previous studies that reported higher mRNA and protein expression of APN in lung tissue cells from older influenza patients compared to younger controls (15), but they contrast with studies indicating lower APN levels in males with COVID-19 infection (17). Our research contributes to the understanding of how LRTI affects APN and AdipoRs in various subgroups defined by sex, age, and BMI, an area that has not been extensively explored in the literature.

In addition, we found that PBMC AdipoR is associated with lipids as well as WBC, lymphocyte and monocyte counts in multivariate regression models in discriminating between LRTI and HC, as well as LRTI subgroups. This illustrates the potential influence of adipokines and routine blood markers of inflammation on PBMC AdipoR, linking metabolism and immunity in tandem (21). It is also interesting to note that among the four lipid profiles (TG, TC, HDL-C, LDL-C), HDL-C showed the greatest significant difference for infections, severe and acute, which may be related to its anti-inflammatory effects (39). It has been shown that in COVID-19, reduced HDL-C is associated with a higher risk of death (40), and patients with low HDL-C on admission are at higher risk of severe infection (41). Reduced HDL-C in the acute group of LRTI may be associated with poor prognosis, which needs further investigation.

The results of the correlation analyses of AdipoR with lymphocyte subpopulations and immunoglobulins in the LRTI group were also interesting, with APN correlated with IgG and IgE, AdipoR1 negatively correlated with absolute NK cell counts and AdipoR2 positively correlated with IgE. It has been shown that APN plays an important role in regulating NK cell function. However, whether APN inhibits or stimulates NK cells remains controversial. An *in vivo* study demonstrated that APN downregulated the frequency of NK cells in the spleen while increasing the efficiency of NK cells (42). Conversely, APN treatment inhibited IL-2-induced cytotoxicity and interferon-γ production in human and mouse NK cells (43). Based on our results, we speculate that APN may be regulating NK cells through AdipoR1. The correlation between IgE and APN is scarce and only seen in a small number of studies of allergic diseases such as asthma (44), and the interaction between AdipoR2 and IgE in infectious diseases may be a future direction of research. Unfortunately, there was some missing information in our study, which limited our further exploration in the subgroups.

This study has some limitations. Firstly, the cross-sectional design does not allow for the identification of causal relationships, and the relatively small sample size may limit the generalizability of our findings. Secondly, we only isolated PBMC without specifically distinguishing between lymphocyte subpopulations and monocytes, and further studies are needed to investigate the changes in AdipoR expression in different lymphocyte subtypes as well as monocytes when LRTI infection occurs. Thirdly, we only isolated PBMC from peripheral blood and did not collect alveolar lavage fluid samples from other sites (e.g., lungs) for some reason. It is still necessary to study the changes of APN in the lung and the expression levels of AdipoRs in lung tissue-resident immune cells during the onset of LRTI to further investigate the immunomodulatory effects of adiponectin signaling.

## 5 Conclusion

Circulating APN and PBMC AdipoR1 mRNA have different detection values in LRTI; PBMC AdipoR1 is correlated with the severity of LRTI, whereas circulating APN levels were significantly elevated in patients with non-acute LRTI. Our results suggest that APN and AdipoR1 (but not AdipoR2) mRNA in peripheral blood mononuclear cells are associated with LRTI and may have a pro-inflammatory effect, which remains to be further investigated.

## Data Availability

The original contributions presented in the study are included in the article/**Supplementary Material**. Further inquiries can be directed to the corresponding author/s.
